# An extensible spatial and temporal epidemiological modelling system

**DOI:** 10.1186/1476-072X-5-4

**Published:** 2006-01-17

**Authors:** Daniel Alexander Ford, James H Kaufman, Iris Eiron

**Affiliations:** 1 Department of Computer Science, IBM Almaden Research Centre, San Jose, CA, 95120, USA

## Abstract

**Background:**

This paper describes the Spatiotemporal Epidemiological Modeller (STEM) which is an extensible software system and framework for modelling the spatial and temporal progression of multiple diseases affecting multiple populations in geographically distributed locations. STEM is an experiment in developing a software system that can model complex epidemiological scenarios while also being extensible by the research community. The ultimate goal of STEM is to provide a common modelling platform powerful enough to be sufficient for all modelling scenarios and extensible in a way that allows different researchers to combine their efforts in developing exceptionally good models.

**Results:**

STEM is a powerful modelling system that allows researchers to model scenarios with unmixed populations that are not uniformly distributed and in which multiple populations exist that are being infected with multiple diseases. It's underlying representational framework, a graph, and its software architecture allow the system to be extended by incorporating software components developed by different researchers.

**Conclusion:**

This approach taken in the design of STEM creates a powerful platform for epidemiological research collaboration. Future versions of the system will make such collaborative efforts easy and common.

## Background

STEM is a computer software system for defining and visualizing simulations of the geographical spread of contagious diseases. It allows simulated scenarios to model multiple population types (e.g., Humans and Ducks) being simultaneously infected by multiple diseases. The ultimate goal of STEM is to provide a common modelling platform powerful enough to be sufficient for all modelling scenarios and extensible in a way that allows different researchers to combine their efforts in developing exceptionally good models.

Prior to STEM, most previous epidemiological modelling systems assumed that a population being affected by a disease was "well mixed" and either not distributed (zero dimensional simulation) or geographically distributed in a uniform manner. This approach has proved productive, but these assumptions are usually not true. Previous approaches also tended to focus on a single population type affected by a single disease. Again, this is not always a true reflection of reality. A topical example where previous approaches fall short is modelling the spread of Avian Influenza and its potential to infect both humans and birds. STEM can model such a scenario.

Previous approaches have also tended to be model specific and not designed for general arbitrary extension by other researchers. The merit in STEM's more general goal of providing a platform that can be extended is that it would allow contributions from different researchers to be leveraged on a common platform. This would reduce duplication, allow models to be compared and refined and enable the strength's of different researchers to be focused on solutions to specific problems, for example, the looming Avian Flu pandemic.

## Results

### Interface and example

A simulation scenario is specified in STEM by the use of a configuration interface (Figure 3). The interface allows for the selection of a geographic model and the specification of one or more population types and one or more disease models. The disease models can be configured by having their parameters altered from their (reasonable) default values. The specification of initial conditions for the simulation such as the "seeding" of infectious population members at locations in the model is also part of the interface.

**Figure 1:**
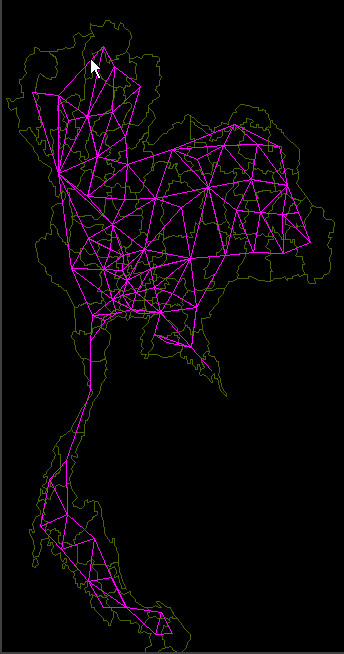
**Graph representation of Thailand. **A map of Thailand with the underlying representational graph showing the "physical adjacency" relationship between locations.

**Figure 2:**
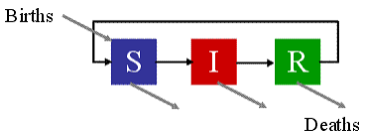
**State diagram for SIR model. **The disease states S, I & R and the allowed transitions between states. All three states have an allowed transition to a "dead" state.

**Figure 3:**
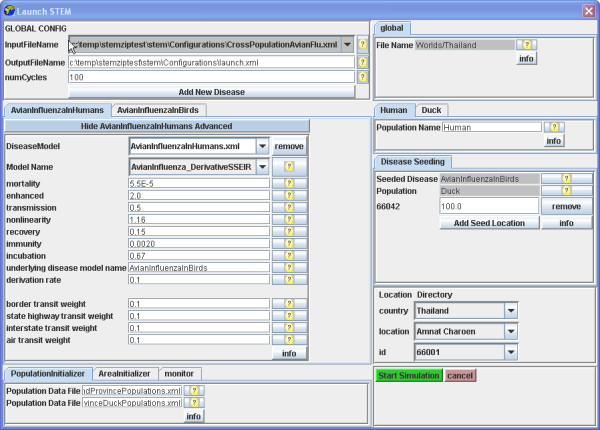
**Configuration interface. **The STEM configuration interface shows the initial setup for modelling Avian Influenza among ducks and humans in Thailand.

When the configuration is complete the user moves on to the simulation display that shows several views of the model and the state of the simulation. The distribution of a population is illustrated by highlighting a geographic area using intensity to indicate relative numbers and colour to indicate disease state. This representation may be adjusted using either logarithmic or linear intensity scales using a varying gain factor. After the simulation is started, the simulation display updates itself after each time step and displays the current state information. Letting the simulation "run" animates the display so that the progression of a disease can be watched in "real-time".

**Figure 4:**
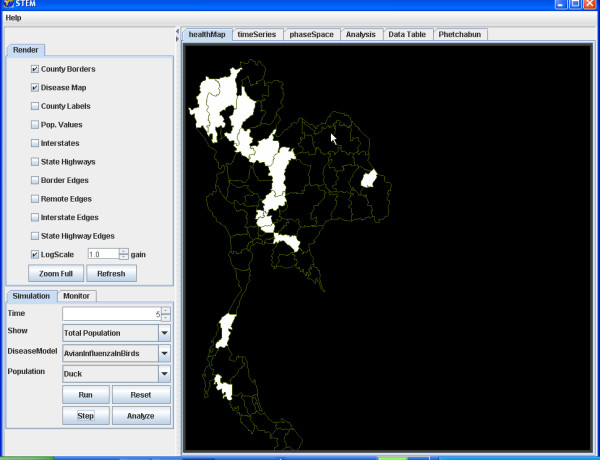
**Distribution of susceptible duck population in Thailand. **The areas highlighted in white show the distribution of a hypothetical susceptible population of ducks in Thailand at the start of a scenario.

**Figure 5:**
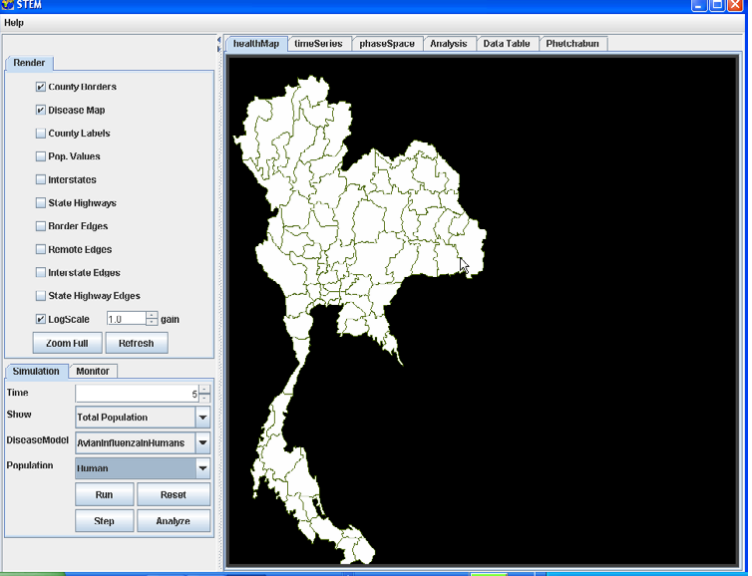
**Distribution of susceptible human population in Thailand. **The areas highlighted in white show the distribution of the susceptible population of humans in Thailand at the start of a scenario.

**Figure 6:**
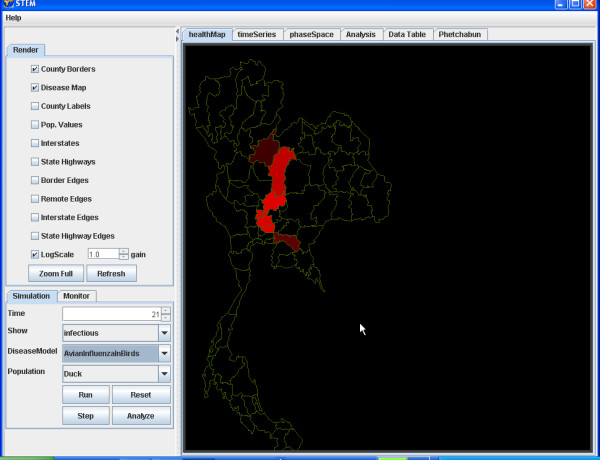
**Geographic distribution infected ducks after 21 simulated days. **The geographic distribution of the population of ducks infected with Avian Influenza in Thailand after 21 simulated days is highlighted by the provinces coloured red.

**Figure 7:**
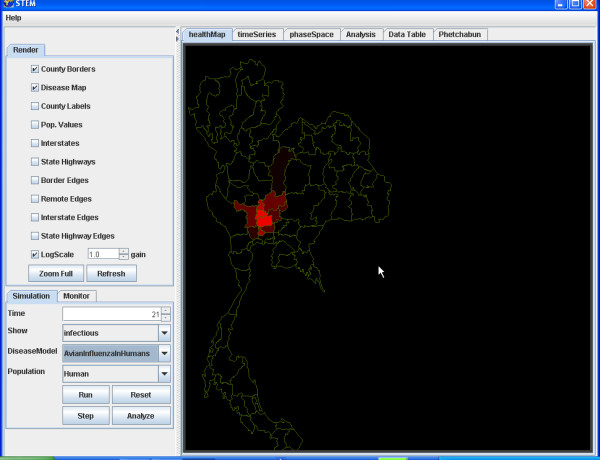
**Geographic distribution infected humans after 21 simulated days. **The geographic distribution of the population of humans infected with Avian Influenza in Thailand after 21 simulated days is highlighted by the provinces coloured red.

**Figure 8:**
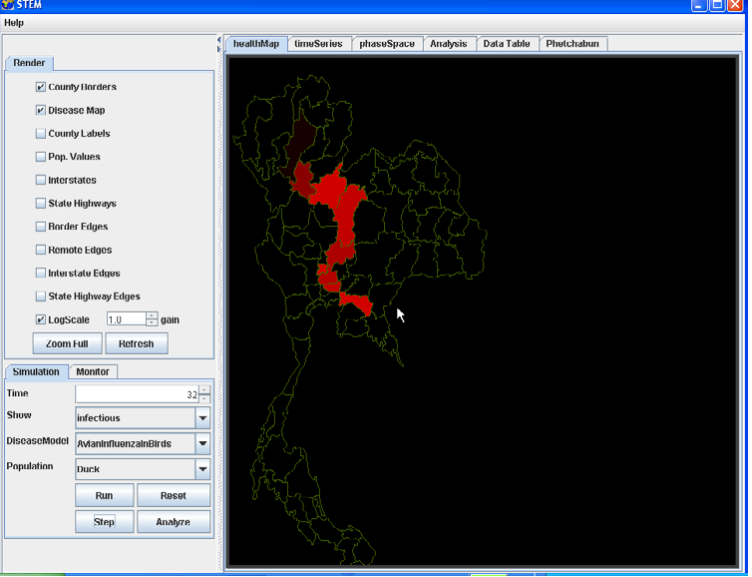
**Geographic distribution infected ducks after 32 simulated days. **The geographic distribution of the population of ducks infected with Avian Influenza in Thailand after 32 simulated days is highlighted by the provinces coloured red.

**Figure 9:**
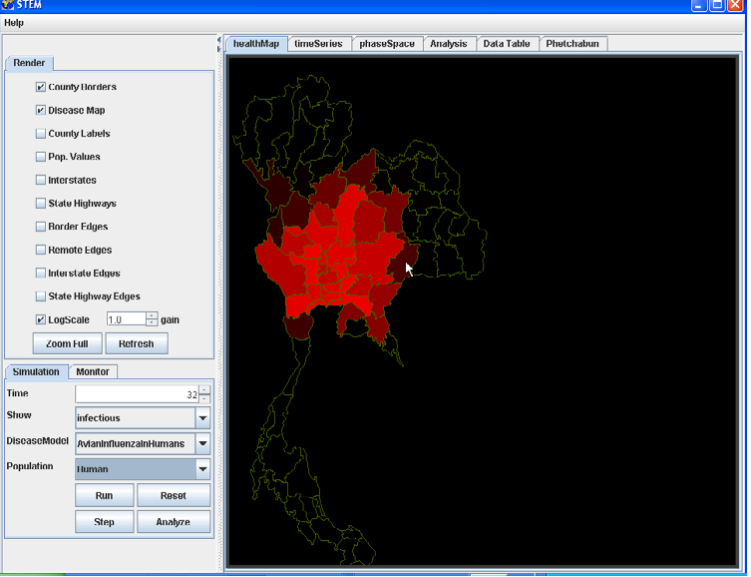
**Geographic distribution infected humans after 32 simulated days. **The geographic distribution of the population of humans infected with Avian Influenza in Thailand after 32 simulated days is highlighted by the provinces coloured red.

For example, we illustrate the simulation of the transmission of Avian Influenza within a hypothetical susceptible population of ducks geographically distributed in Thailand (Figure 4) and to the susceptible population of Humans in Thailand (Figure 5). The simulation begins with a seeding of one hundred infectious ducks in the province of Phetchabun. After twenty-one days the disease has spread to ducks in adjacent provinces (Figure 6) and to Humans (Figure 7). After thirty-two days, most of the duck population has been infected (Figure 8) and the geographic spread of the disease to the human population has greatly expanded (Figure 9).

## Discussion

### Related work

Other research that exploits graph theory to create models of complex scenarios includes representing of the movements of pigs [1] in Sweden over a six month period to model swine fever. Graph Theory has also been used [2] to model the spread of mycoplasma pneumonia among patients in a hospital. In that model, the patients were vertices and edges represented "contact" between them.

Other approaches to disease simulation have also been successful. Discrete or "agent based" simulations have been developed that model the state of individual population members and track their movements in a simulated world. These "agents" walk on streets, ride buses, get on elevators and do other detailed activities. As they interact in the simulated world they spread the modelled disease. A good example of such a sophisticated system is EpiSims which has been used to model an outbreak of Small Pox in the city of Portland, Oregon [3]. The advantage of this approach is that it promises to give accurate and understandable results. The disadvantage is that it requires large amounts of computing resources even for small models. Attempting to simulate large geographic scenarios with large populations is very expensive and takes long periods of time.

The advantage of a system using STEM's design is that it can produce meaningful results faster and with fewer resources. The resolution of the underlying graph can be adjusted to model greater detail by using more nodes to represent specific areas. For instance, one could model North America with Mexico and Canada represented at the state/province level (tens of nodes) while the United States is represented at the county level (over three thousand nodes).

### Hybrid models

The representational framework in STEM opens up the possibility of creating hybrid continuous/discrete simulation models. The advantage of this would be the ability to model large geographic areas, such as all of Earth, by using continuous modelling (i.e., what STEM does now) for large areas and agent based systems for smaller more specific areas like a city. In STEM, the labels on a node that represent disease state and the computations that change them can be supplied by external software components that could act as proxies to external simulation systems. Thus, STEM could "glue" together a hybrid model in which a number of different parallel agent based simulations model specific cities and STEM models everywhere else.

## Conclusion

STEM demonstrates that it is possible to create an epidemiological simulation system that is both powerful and extensible. The use of a graph as the representational framework is a powerful technique. It allows arbitrary variations in geographic resolution and makes it possible for STEM to represent a non-uniform population distribution.

STEM's extensible architecture also demonstrates that with appropriate software design, it is possible to create a collaborative modelling platform. Such a platform could form the basis of a common modelling infrastructure that would leverage the efforts and expertise of researchers in many domains (e.g., Epidemiology, Mathematics, Geography, Orthonology, and Computer Science) to create accurate simulation scenarios for diseases that will affect us all.

## Methods

### Representational framework

The internal representation framework used in STEM is a graph [4]. This is a powerful mathematical abstraction that has been applied to many different problem domains. STEM uses the graph to represent geographic locations and their relationships. In a graph a *node *represents a geographic location. Depending on the resolution, nodes in a particular graph may represent a country, state, county or city. It is even possible to combine graphs with different resolutions so some regions may be modelled with higher resolution than others. An *edge *in a graph represents a relationship between two geographic locations (Figure 1). There are many kinds of relationships that could exist. These include "physical adjacency", "linked by state highway", or "exchange population via air travel". Each such relationship is characterized by a metric value that represents the "weight" of the relationship, for instance, the average number of people who travel between two locations (nodes) in a day. This weight value can be incorporated into the disease model computations to account for the level of contact between populations in different geographic locations.

This framework negates the need for typical epidemiological modelling assumptions of a "well mixed" uniformly distributed population. This allows for more realistic models to be represented. The framework also allows for geographic representations at different levels of resolution so that models can focus on key areas without excluding the importance of neighbouring regions. The ability to incorporate abstract relationships in the framework between geographic locations further enhances STEM's ability to model complicated scenarios.

### Disease model state representation

STEM represents the state of a disease affecting a population at a geographic location as a *label *on the corresponding node in the representational graph. Typically, the label segments the population into different discrete disease states and records the numbers of population members in each state. An SIR model [5] has states of *susceptible*, *infectious *and *recovered *(Figure 2), an SEIR model includes an *exposed *state and an SI model removes the recovered state. More than one label may be attached to a node so the states of several diseases and several populations can be maintained simultaneously at each geographic location.

In the SIR state model, for example, a population member can be born and is instantly susceptible and so enters the state S, they can then either stay in that state, become infectious and move to state I, or they can die. In state I, a population member can stay in state I, or recover and move to state R, or die. Similarly, in state R, they can stay in R, lose their immunity and move to state S, or die. The transition from R to S will not be present in all models as it represents population members losing their immunity to a disease which will not be the case for all diseases.

### Computations

The disease model computations implemented in the base version of STEM are rate equations that specify the number of population members that enter and leave each of the representational disease states (e.g., S, I or R) for a particular interval of time.

STEM comes with four "built-in" disease models corresponding to the SIR and SEIR disease models and stochastic or deterministic computations (2 × 2). It also includes a unique disease model for multi-serotype diseases such as dengue fever which maintains a combinatorial disease state that tracks the sequential serotype infection and recovery state for all possible sequence of infections [6].

### Simulation

The simulation of the progression of a disease is implemented in STEM as an iterative process that visits each node in the graph and computes a new value for each disease state label for each population at that node. The new values are computed solely from the current state of the simulation, which consists of the current simulated time and the current values of the labels on the nodes in the graph. The new values are saved as they are computed and only when all nodes have been so processed at the end of the cycle do the new labels replace the previous labels. Simulation time is then advanced by the time delta of the simulation (typically a day, but this is configurable). The process repeats until stopped by the user. As each cycle is processed, history data is collected for the disease states so that the progress of the disease can be reviewed or analyzed later.

### Implementation and extensibility

STEM is written in the Java™ programming language and runs on most platforms that support Java™. The architecture is the system is designed to be extended by the addition of new disease models. This involves the writing of software modules in the Java™ programming language. The current base distribution of STEM comes complete with example source code for all its disease models. These models can be augmented by user supplied implementations of additional disease models. A direct approach to doing this is to copy the sample code and modify a renamed version. This allows for simple changes to the base calculations to be developed quickly and incorporated for general use. More extensive changes require deeper understanding of how to manipulate the representational framework (documented in the distribution), but allowing such changes was one of the design requirements for the system so this type of extension is deliberately exposed to other researchers.

In a future version of STEM, the code base will be retargeted to the eclipse open source tool framework [7]. This framework provides a formal "plug-in" software architecture that will be leveraged by STEM to provide a more standardized approached to its extension. This will allow not just disease models to be extended, but also features such as new and different types of graphic displays and other interface items as well as the modelling of more and different relationships between locations.

## Authors' contributions

DAF designed the architecture for STEM and implemented the core simulation engine, representational framework and disease models and drafted the manuscript. JHK developed the technique for aggregating the contribution of infectious population members at related locations into the computations at a specific location, collected the geographic data, specified most of the modelling scenarios that come with the system, developed the user interface and participated in the draft of the manuscript. IE developed an early prototype of STEM. All authors read and approved the final manuscript.
